# Low-Concentration Atropine Monotherapy vs. Combined with MiSight 1 Day Contact Lenses for Myopia Management

**DOI:** 10.3390/vision6040073

**Published:** 2022-12-12

**Authors:** Nir Erdinest, Naomi London, Itay Lavy, David Landau, Dror Ben Ephraim Noyman, Nadav Levinger, Yair Morad

**Affiliations:** 1 Department of Ophthalmology, Hadassah-Hebrew University Medical Center, Faculty of Medicine, Hebrew University of Jerusalem, Jerusalem 9574409, Israel; 2 The Myopia Center, Rishon LeZion 4951732, Israel; 3 Naomi Vision Boutique, 5 Even Israel St., Jerusalem 9422805, Israel; 4 Department of Ophthalmology, Rambam Health Care Campus, Haifa 3525408, Israel; 5 Department of Ophthalmology, Enaim Refractive Surgery Center, Jerusalem 9438307, Israel; 6 Department of Ophthalmology, Assaf Harofeh Medical Center, Zerifin 7033001, Israel

**Keywords:** myopia, myopia progression, myopia control, atropine, contact lenses

## Abstract

Objectives: To assess the decrease in myopia progression and rebound effect using topical low-dose atropine compared to a combined treatment with contact lenses for myopic control. Methods: This retrospective review study included 85 children aged 10.34 ± 2.27 (range 6 to 15.5) who were followed over three years. All had a minimum myopia increase of 1.00 D the year prior to treatment. The children were divided into two treatment groups and a control group. One treatment group included 29 children with an average prescription of 4.81 ± 2.12 D (sphere equivalent (SE) range of 1.25–10.87 D), treated with 0.01% atropine for two years (A0.01%). The second group included 26 children with an average prescription of 4.14 ± 1.35 D (SE range of 1.625–6.00 D), treated with MiSight 1 day dual focus contact lenses (DFCL) and 0.01% atropine (A0.01% + DFCL) for two years. The control group included 30 children wearing single-vision spectacles (SV), averaging −5.06 ± 1.77 D (SE) range 2.37–8.87 D). Results: There was an increase in the SE myopia progression in the SV group of 1.19 ± 0.43 D, 1.25 ± 0.52 D, and 1.13 ± 0.36 D in the first, second, and third years, respectively. Myopia progression in the A0.01% group was 0.44 ± 0.21 D (*p* < 0.01) and 0.51 ± 0.39 D (*p* < 0.01) in the first and second years, respectively. In the A0.01% + DFCL group, myopia progression was 0.35 ± 0.26 D and 0.44 ± 0.40 D in the first and second years, respectively (*p* < 0.01). Half a year after the cessation of the atropine treatment, myopia progression (rebound effect) was measured at −0.241 ± 0.35 D and −0.178 ± 0.34 D in the A0.01% and A0.01% + DFCL groups, respectively. Conclusions: Monotherapy low-dose atropine, combined with peripheral blur contact lenses, was clinically effective in decreasing myopia progression. A low rebound effect was found after the therapy cessation. In this retrospective study, combination therapy did not present an advantage over monotherapy.

## 1. Introduction

Myopia is the most common refractive error in the world and a leading cause of vision loss [1,2]. A recent study estimated that the prevalence of myopia between the ages of 8–16 in the United States is currently approximately 16.5% [3]. The prevalence of myopia is expected to increase, and it is estimated that, by the year 2050, myopia will affect approximately half the population in the Middle East as well as the world’s population, and high myopia (5.00 diopters (D) and above) will affect about 10% of the world’s population [4].

In those with myopia, the cornea’s refractive power or the eye’s length is too powerful, causing the light rays to focus in front of the retina. When this deficiency begins to develop from the age of six to ten, it tends to progress at a more rapid rate. Adults with lower refractive errors tend to stabilize, though not absolutely, around the age of 15–18 [5,6,7].

Myopia is a disease with severe implications in terms of treatment costs and possible eye complications, including glaucoma, retinal holes, tears, and detachments, and even the appearance of strabismus and double vision [8,9,10]. Myopia is considered one of the leading causes of blindness in the western world and Israel as well [11]. Myopia is Israel’s third most common cause of blindness after age-related macular degeneration and glaucoma [8,9,10,12].

Many theories have been linked to the causes of myopia progression. Beyond the genetic component, a lack of exposure to natural daylight (many children spend very little time outside) and an abundance of short-range work (reading or using digital screens) contribute to the progression [3,13,14].

Among the tools currently available to try and manage myopia, orthokeratology contact lenses, dual-focus contact lenses, and multifocal contact lenses with a far-center design have exhibited moderate to considerable success [15].

The most effective treatment to date, as shown in research studies, is atropine drops, though it seems to be influenced by individual characteristics (for example, age, genetic makeup, and degree of myopia) and is dose-dependent. Although atropine at high concentrations (1% and 0.5%) achieved a higher efficacy at slowing myopia progression, a low concentration of atropine, such as 0.01% and particularly 0.05%, was found to be similarly effective, with the advantage of a minimal effect on accommodation and pupil dilation, and less of a rebound effect (high increase in myopia after stopping treatment) [1,11,16].

Topically administered atropine is believed to operate on several fronts to control myopia. First, as a muscarinic antagonist, it slows down the rate of eye elongation and affects the processes of the growth of the sclera. In addition, atropine affects the release of cellular dopamine and, therefore, influences the retinal signals that control the growth rate of the eye [8,9,17].

Topical atropine treatment is commonly prescribed in low concentrations, such as 0.01% and 0.05% atropine, the latter of which is currently discussed in the published literature as the preferred concentration [1,11,16,17,18]. Atropine at such a low concentration has many advantages, including a minimal effect on pupil size (less than 0.8 mm), minimal loss of accommodation (1.5 diopters on average), and less loss of near vision compared to higher concentrations [1,11,16,17,18]. The results were also supported by another study in which it was found that atropine at a concentration of 0.01% significantly reduces the rate of myopia progression over a year with minimal side effects [1,11,16,17,18]. It should be noted that, for myopia with a rapid progressive nature, higher concentrations of atropine treatment are required [1,11,16,17,18]. Multicenter studies have also shown the effectiveness of atropine treatment. After three years, the axial length increased significantly less than the control group, causing glare and requiring near-work spectacles [1]. A multiparticipant study treated with atropine concentrations of 0.5%, 0.1%, and 0.01% showed a significant slowing in myopia progression compared to children treated with saline [19]. Children treated with the low concentrations of atropine did not complain of blurred near vision or glare, unlike those treated with atropine at the high concentration [19]. However, three years after the end of the study and the cessation of treatment, the children treated with high concentrations suffered from an increased rebound phenomenon compared with the children treated with low concentrations [19]. The professional literature has shown that the combination treatments in some challenging cases can often be more effective than atropine alone, such as atropine and orthokeratology (ortho-k) contact lenses treatment, or atropine and bifocal spectacles maintain a decreasing axial growth [1,20,21,22].

At the same time, studies have found that retinal peripheral hyperopic blurring is a leading factor in axial length increase, hence the importance of controlling peripheral refraction [1,17,23]. Peripheral refraction control contact lenses are contact lenses with a double-focus point similar to prescription contact lenses for vision correction, where the peripheral addition reduces the relative hyperopia caused by a monofocal distance lens. Unlike spectacles, where the correction for near vision is only in the inferior area of the lens, in a contact lens, the addition for near vision is in the entire circumference of the lens [1,17,23]. The center of the lens corrects the distance vision, while at the periphery, the power of the lens decreases gradually toward the periphery (in a concentric design), by approximately 2.00–3.00 D. These lenses were found to slow myopia progression by approximately 50% compared to spectacles [1,17,23]. Recently, a published study found these lenses to be effective for three years and have a high safety profile [1,17,23].

## 2. Materials and Methods

The study included 85 Caucasian children with a myopic refractive error. The clinic that conducted this study primarily treats patients specifically interested in myopia control. The treatments are determined using multiple considerations including the severity of myopia, the rate of progression prior to consultation, and the willingness of the child and their caregivers to wear contact lenses. In general, the children who present with a history of rapid myopia progression, particularly the younger aged children, and the children and parents who consent to wearing contact lenses will be encouraged to receive combined treatments, yet it was not always accepted. The average age was 10.3 ± 2.2 (range 6 to 15.5), and the spherical equivalent (SE) value was 4.69 ± 1.8 D (Range −1.25 to −10.87 D) at the beginning of the treatments.

The children were divided into two treatment groups and a control group, which included 30 children who wore single-vision spectacles (SV), averaging −5.06 ± 1.77 D, ranging in a sphere equivalent (SE) from −2.37–8.87 D. The first study group included 29 children with an average SE prescription of −4.81 ± 2.12 D (range −1.25–10.875 D), who were treated with 0.01% atropine for two years (A0.01%) and followed up to one year after that. The second group included 26 children with an average SE prescription of 4.14 ± 1.35 D (SE range of 1.625–6.00 D) and who were treated with MiSight^®^ 1 day (Cooper Vision, Pleasanton, CA, USA) contact lenses with dual focus (DFCL) and 0.01% atropine (A0.01% + DFCL) for two years and followed for one year after that. The children all had a minimal increase in myopia of 1.00 D during the year prior to treatment. The children’s demographics are summarized in Table 1.

After two years of treatments, the first and second groups stopped myopia management, and an SE comparison was made to the SV group at the end of the third year.

### 2.1. Ethical Principles

This study followed the tenets of the Helsinki Declaration. The Medical Center Institutional Review Board (IRB) approval was obtained for this study (HMO-0354-21), and all procedures were carried out per their guidelines. The parents were aware that their children were participating in this study.

### 2.2. Inclusion and Exclusion Criteria

Inclusion criteria included both a cycloplegic spherical equivalent refraction (SER) equal to or above −1.00 D in each eye and a best-corrected visual acuity of 6/9 or superior. The children had a myopic progression documented in their file of at least −1.00 D during the year prior to beginning treatment.

Children with systemic or ocular diseases, such as connective tissue disorders, strabismus, or any previous atropine therapy (for myopia progression or amblyopia), were excluded. Children with astigmatism greater than 2.00 D, and anyone that had experience with rigid gas permeable contact lenses, including orthokeratology lenses, were excluded. Anyone that habitually wore spherical or astigmatic soft contact lenses ceased lens wear for two or four weeks, respectively, before commencing treatment.

### 2.3. Treatment Components

The preparation of atropine sulfate 0.01% was provided by a chain pharmacy (Super-Pharm Professional, Petach-Tikva, Israel). The drops were packaged in opaque bottles to prevent photodegrading. The sterile bottle size was 10.0 mL, consisted of 5 mL of preparation preserved with Benzalkonium chloride 0.01%. The bottles were stored at 4 °C for no longer than 21 days. The parents of the appropriate groups were instructed to instill one drop daily before bedtime.

The soft contact lenses prescribed were MiSight^®^ 1 day (Cooper Vision, Pleasanton, CA, USA) containing 60% water and 40% Omafilcon A, hydrogel contact lens material (non-ionic) for daily wear single use. The lens has a total diameter of 14.2 mm, comprising an 11.66 mm optic zone with four alternating distance and near prescription zones (maximum treatment zones with an addition of +2.00 diopters). The central distance optic zone is 3.66 mm in diameter. Children were instructed to wear the lenses daily for eight hours a day.

### 2.4. Follow-Up Visits

The children were examined bi-annually throughout the three years of the study (including a one-year washout). Refraction was measured at each visit with two instillations of 1% tropicamide, one drop instilled in each eye at 5-min intervals. Subjective refraction was performed post-cycloplegia approximately half an hour after instillation of the second drop by a single practitioner in the same examination room. The children wore the correction modality prescribed at the beginning of the study (bifocals, progressive added lenses, contact lenses). Distance visual acuity (VA) was measured monocularly at each visit with the same Snellen chart and identical ambient lighting. The optical devices were changed as required after each visit to the newly measured refraction.

### 2.5. Cessation of Atropine Therapy

Atropine therapy cessation was conducted gradually under detailed guidelines to reduce possible subsequent rebound [24]. This study implemented the discontinuance protocol at the end of two years.

The number of atropine instillation days per week was reduced monthly (one day per week per month) over the course of six months. During the first month post-treatment, the patient would instill atropine six days per week, five days per week during the second month. During the third month, four days per week (every other day), and during the fourth month, days one, three, and five. During the fifth month, on days one and four and during month six, once a week.

A cycloplegic refraction and retinal exam were performed at the end of months seven, and six months later.

### 2.6. Statistical Analysis

Analysis of the myopia progression for each group, between the groups including age and refractive error, was performed using the Statistical Package for Social Sciences software 25.0 (SPSS Inc., Chicago, IL, USA) with One-way Analysis of Variance (ANOVA) and Tukey-Kramer Multiple Comparisons Tests.

## 3. Results

**Figure 1:**
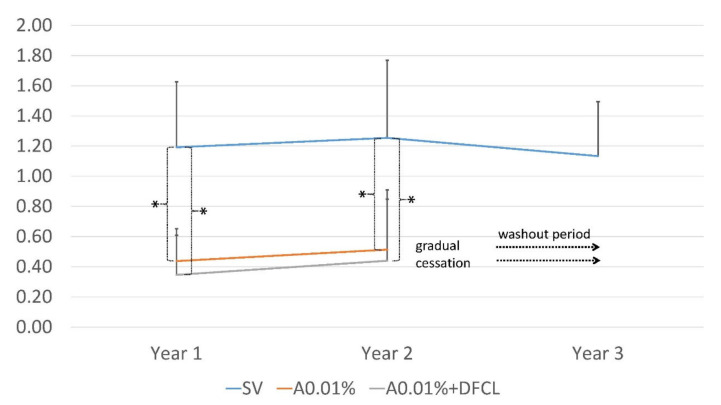
Increase in myopia over three years in the group wearing single-vision spectacle lenses (SV), two years of treatment with 0.01% atropine (A0.01%), and two years wearing dual-focus contact lenses (DFCL) with A0.01% (A0.01% + DFCL). One asterisk (*) represents a significant *p* value (*p* < 0.01). SV: single-vision spectacle lenses. A0.01: 0.01% atropine for two years of treatment. A0.01% + DFCL: contact lenses with dual focus and topical 0.01% atropine for two years.

There was an increase in the SE myopia progression in the SV group of 1.19 ± 0.43 D, 1.25 ± 0.52 D, and 1.13 ± 0.36 D in the first, second, and third years, respectively. The myopia progression in the A0.01% group was 0.44 ± 0.21 D (*p* < 0.01) and 0.51 ± 0.39 D (*p* < 0.01) in the first and second years, respectively. In the A0.01% + DFCL group, the myopia progression increased by 0.35 ± 0.26 D and 0.44 ± 0.40 D in the first and second years, respectively (*p* < 0.01). The total progression over the three years in the SV group was 3.57 D (average 1.19 ± 0.43 D annually), in the A0.01% group 1.196 D (average 0.34 ± 0.34 D annually), and in the A0.01% + DFCL group 0.96 D (average 0.324 ± 0.33 D annually) (Figure 1).

**Figure 2:**
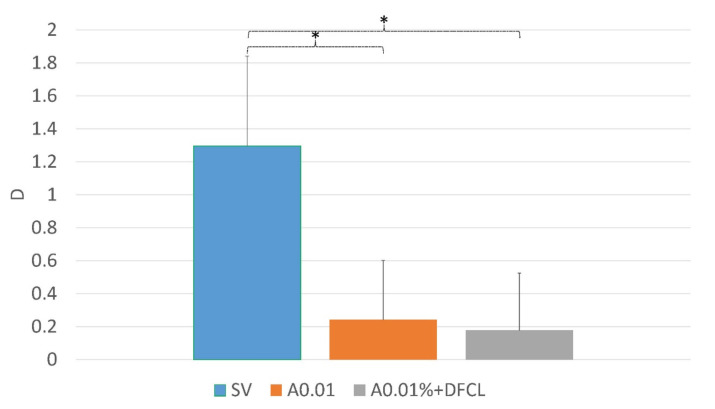
The rebound effect was tested one year after the cessation of treatment in the SV group, atropine at a concentration of 0.01% (A0.01) and after contact lenses with dual focus (DFCL) together with 0.01% atropine (A0.01% + DFCL). One asterisk (*) represents a significant *p* value (*p* < 0.01).

Half a year after the cessation of the atropine treatment, the myopia progression (rebound effect (was −0.24 ± 0.35 D and −0.18 ± 0.34 D in the A0.01% and A0.01% + DFCL groups, respectively (Table 2, Figure 2).

## 4. Discussion

This study shows the effectivity of atropine therapy as a stand-alone treatment and combined with peripheral defocus lenses, and the low rebound effect when atropine is tapered following the treatment.

### 4.1. Method Strengths and Weaknesses and Rebound

Refractive error measurements were performed under cycloplegia. Cycloplegia was achieved with tropicamide, not cyclopentolate, which may raise some questions. Although cyclopentolate is the more prevalent and, in some cases (particularly hyperopes), preferred as it has been shown to produce more effective cycloplegia, trials have shown equal effectiveness between the two, and this was the preferred method of these caregivers [6].

Though many studies prefer the high-resolution availability of the autorefractor to monitor the myopia progression, a subjective cycloplegic refraction is the preferred method of many clinicians. It is considered the “gold standard” for accurate refraction and spectacle prescription rather than automated or retinoscopic objective refractions [25]. Though some autorefractors are more accurate than others, some tend to stray even further from the subjective with cycloplegia, and further from the subjective than retinoscopy [26,27], which is generally very close to the subjective when performed by a skilled clinician [26,27].

There is a concentration-dependent response for low-concentration atropine, as shown in the Low-concentration Atropine for Myopia Progression (LAMP) [28]. Furthermore, the child’s age plays a vital role on two levels. First, a young age is associated with the need for a higher concentration to achieve high efficacy [29], and, second, younger children tend to have a faster myopic progression [29].

Even at the same concentration and within the same age group, there are still variations in treatment responses [29]. The current study showed a relatively large age diversity, which may be considered a limitation, with the control group (SV) containing the lowest age and the A0.01% + DFCL group containing the highest age. However, there was a similar range between the groups and, between the treatment groups, there was no statistical difference in the myopia progression (or lack thereof). Both were significantly effective at decreasing previous myopia progression.

Younger children treated with lower concentrations showed a rebound effect similar to older children treated with higher concentrations [30]. The LAMP study further suggested that low-concentration atropine treatment in children should be ended at older ages when both the natural myopic progression rate and the rebound effect become smaller [30]. The World Health Organization has begun suggesting that treatment cessation for one year should be considered if good treatment responses are observed after two years of continuous therapy, and those who show progression after the one-year cessation can be offered further treatment [1]. While no study, as yet, can precisely quantify the number of years of treatment that would be appropriate, the literature is starting to examine the tolerance, safety, and effectivity of treatment lasting three years or longer [23,30,31].

While younger children may require 0.05% atropine to achieve adequate control over progression, this concentration often induces significantly more mydriasis and loss of accommodation amplitude than 0.01% atropine in Caucasian children who may be less tolerant of the side effects. In addition, it is important to note that a greater rebound effect was associated with a 0.05% atropine concentration (compared to 0.01%) in the younger age group at the treatment cessation [28], and, in this cohort, the lower concentration proved effective.

At the time these children were treated, the predominant and preferred concentration in the published literature was 0.01%. The advantage of the 0.05% concentration, particularly in younger children who may be less affected by the 0.01% concentration, was published later. When the 0.01% concentration proves insufficient, one option is to combine the treatment with another that will assist from a different approach, for example, peripheral defocus, to reduce the hyperopic shift in the retina’s periphery. The combined therapy in the group here uses a contact lens with two treatment zones which have been shown to be effective as a monotherapy and add effectivity in a case series [32].

### 4.2. Peripheral Defocus Design

The trial conducted by Anstice and Philips showed that eyes wearing MiSight (Cooper Vision, Pleasanton, CA, USA) contact lenses had significantly less axial elongation than eyes wearing single-vision lenses [33]. Following the above study, Chamberlain et al. conducted a multicenter study in several countries over three years. The refractive error progression in the first year was 0.40 D less compared to the control group. In the second year, the progression was 0.54 D less and, in the third year, 0.73 D less than the control group. The axial elongation change was as follows: at 12 months, the axial length change in the control group was 0.24 mm compared to 0.09 mm in the MiSight group. At 24 and 36 months, the axial length change was 0.24 and 0.32 mm, respectively, less than the control group. Regarding refraction, the myopia control effect after three years was 59%, and, in the axial length control, the effect was a 52% decrease [34].

The Bifocal Lenses In Nearsighted Kids (BLINK) study, which began in 2017, compared identical design single-vision and center distance soft multifocal contact lenses with a +1.50 D addition to a +2.50 D addition [35,36]. The results so far suggest that there may be either a dioptric threshold or a minimal area of visual field blur necessary to achieve the inhibitory effect [1]. This information supports using a lens such as the MiSight, which incorporates two concentric circles of peripheral blur, thereby providing a larger retinal area of peripheral defocus and assuring blur in both photopic and scotopic environments.

The precise mechanism underlying the positive influence of optical peripheral myopic defocus is not understood. Hypotheses include reducing the accommodative lag [37] and possibly suspending Bruch’s membrane’s excessive expansion [38]. Still undetermined is the exact location on the retina, the surface area required, or the depth of myopic defocus required for maximum efficacy.

Orthokeratology (Ortho-K) lenses are rigid contact lenses with a design profile of reverse geometry [15,39]. This method puts a contact lens on the eye while sleeping at night [15,39], effectively decreasing the myopia progression by using a similar mechanism of peripheral defocus [1,15,39]. It is also sometimes used in combination with low-dose atropine. Studies that compared the effects of Ortho-K and atropine in myopia control showed that Ortho-K treatments might sometimes be superior to a very low atropine concentration (0.125% and 0.02%) in inhibiting the axial length in children with high myopia [40,41]. A combined treatment of 0.01% atropine and Ortho-k can provide better efficacy than monotherapy, as shown in studies over one and two years of treatment [16]. A recent study further analyzed and discovered an advantage of the Ortho-K effectivity in the younger cohort [16], which, when considering the need for a slightly higher atropine concentration in young children, as discussed earlier, may encourage the use of combination treatments of peripheral defocus and a slightly higher concentration of atropine (0.02% or 0.05%) in a young cohort with multiple parameters possibly contributing to a substantial rapid progression for maximum control.

Gradual tapering of the atropine therapy induced a low rebound effect in this cohort, and the combination therapy did not influence it.

### 4.3. Limitations

This study’s limitations are partially inherent within its retrospective nature, in order to assure similar demographics between the groups. While contact lenses may have been recommended to other patients with similar baseline characteristics, they do not always accept the clinician’s proposal. Additionally, a larger cohort would have been preferable. The three groups in this study were comparable in age and refractive error range; however, the wide range of ages within each group may affect the significance of the results as the effectiveness of the treatment is also impacted by the age of the myopia onset [1,9,11].Furthermore, binocular vision measures were not recorded; binocular vision disorders, including esophoria, accommodation lag, and high accommodation versus convergence ratio may influence myopia progression [1,11]. The most effective addition in the periphery of a contact lens and the desired area of the pupil area required to reduce peripheral hyperopia is still being researched. As these data emerge and are implemented, the treatment effects may differ. The authors acknowledge the importance of documenting eye length in the progression of myopia, which is the primary reason treatment is initiated. While changes in the corneal and deceleration of lens power loss of the crystalline lens can contribute, to date, the axial length is still noted to be the defining contributor to myopia progression [42,43]. A limitation to this study is the lack of axial length measurements, which were unavailable, but, as axial length measurements are far more sensitive than refraction, the low myopia progression in this cohort can be reassuring.

In addition, it has been known for a long time that environmental factors contribute to myopia development. In recent years, evidence has accumulated that activity outside the home in daylight effectively delays myopia onset [44]. This information is relevant to the group of children in the study who wore contact lenses and were also treated with atropine, as they may be more active and spend more time outside than those who wore spectacles [45]. If this hypothesis is correct, the study may have been biased because of sunlight’s positive effect on myopia progression [46].

## 5. Conclusions

Monotherapy low-dose atropine, combined with peripheral blur contact lenses, was clinically effective in decreasing myopia progression. A low rebound effect was found after therapy cessation. Combination therapy did not present an advantage over monotherapy in this retrospectively selected cohort.

## Figures and Tables

**Table 1:** Children demographics. SV: single-vision spectacle lenses (control group). A0.01: 0.01% atropine for two years of treatment. A0.01% + DFCL: contact lenses with dual focus and topical 0.01% atropine for two years. VA = visual acuity; SD = standard deviation; SE = spherical equivalent.

	Groups	SV	A0.01%	A0.01% + DFCL
	n	30	29	26
	Gender	53% male	51% female	62% female
Age	Avg and SD	9.08 ± 2.31	10.93 ± 1.94	11.12 ± 1.99
	Range	9–15.5	8–15	9–15.5
SV vs. A0.01%	*p* < 0.01			
SV vs. A0.01% + DFCL	*p* < 0.01			
A0.01% vs. A0.01% + DFCL	*p* > 0.05			
VA and SD	(LogMar)	0.055 ± 0.065	0.097 ± 0.07	0.171 ± 0.259
SE (D)	AVG	5.06	4.81	4.14
	SD	1.77	2.12	1.35
	Min	2.375	1.25	1.625
	Max	8.875	10.875	6.00
SV vs. A0.01%	*p* > 0.05			
SV vs. A0.01% + DFCL	*p* < 0.05			
A0.01% vs. A0.01% + DFCL	*p* > 0.05			

**Table 2:** Myopia progression during three years of the study SE: spherical equivalent, SV: single-vision spectacle lenses. A0.01: 0.01% atropine for two years of treatment. A0.01% + DFCL: contact.

	Myopia Progression SE (D)		
Groups	Year 1	Year 2	Year 3
SV	1.19 ± 0.43	1.25 ± 0.52	1.13 ± 0.36
A0.01%	0.44 ± 0.21	0.51 ± 0.39	0.24 ± 0.35
A0.01% + DFCL	0.35 ± 0.26	0.44 ± 0.40	0.18 ± 0.34
SV vs. A0.01%	*p* < 0.001	*p* < 0.001	*p* < 0.001
SV vs. A0.01% + DFCL	*p* < 0.001	*p* < 0.001	*p* < 0.001
A0.01% vs. A0.01% + DFCL	*p* > 0.05	*p* > 0.05	*p* > 0.05

## Data Availability

The data is available upon request from the corresponding author.
